# Harnessing immunological targets for COVID-19 immunotherapy

**DOI:** 10.2217/fvl-2021-0048

**Published:** 2021-08-09

**Authors:** Abhishesh Kumar Mehata, Matte Kasi Viswanadh, Vishnu Priya, Vikas, Madaswamy S Muthu

**Affiliations:** ^1^Department of Pharmaceutical Engineering & Technology, Indian Institute of Technology (BHU), Varanasi, 221005, India

**Keywords:** COVID-19, cytokine storm, immunotherapy, innate immunity, novel coronavirus

## Abstract

COVID-19 is an infectious and highly contagious disease caused by SARS-CoV-2. The immunotherapy strategy has a great potential to develop a permanent cure against COVID-19. Innate immune cells are in constant motion to scan molecular alteration to cells led by microbial infections throughout the body and helps in clearing invading viruses. Harnessing immunological targets for removing viral infection, generally based on the principle of enhancing the T-cell and protective immune responses. Currently-approved COVID-19 vaccines are mRNA encapsulated in liposomes that stimulate the host immune system to produce antibodies. Given the vital role of innate immunity, harnessing these immune responses opens up new hope for the generation of long-lasting and protective immunity against COVID-19.

In Wuhan, Hubei Province, China, many severe acute respiratory cases were reported with co-existing pneumonia in December 2019, which were later confirmed to be caused by SARS-CoV-2 [1]. The ongoing pandemic in 2019–21 was led by the global spreading of novel coronavirus referred to as COVID-19. The commonly observed pathological symptoms of COVID-19 include elevated body temperature, cough and breathing problems. Other generalized symptoms that may appear are muscular algia, dehydration, diarrhea, dry cough and abdominal pain. The majority of patients have been detected with mild symptoms, but few of them may have progressed to severe pneumonia with vital-organ failure [2,3].

Severe acute respiratory syndrome (SARS) and Middle East respiratory syndrome (MERS) endemics were also caused by viruses of the Coronaviridae family. SARS-CoV endemic has emerged from Southern China in 2003 with 8098 reported cases and 774 deaths globally and, the fatality rate was estimated to be 14–15%. MERS was initially reported in Saudi Arabia in the year 2012 with 2494 confirmed cases and 858 deaths with a high fatality rate of around 34%. While the number of SARS-CoV infections has diminished since 2004, MERS-CoV again appeared in 2012 which has resulted in multiple sporadic outbreaks in numerous countries [3–5]. As SARS-CoV-2 is recently identified, detailed immunological information about COVID-19 is yet to be established such as the nature of viral epitopes eliciting antibody formation and T-cell responses [4]. Currently, the ongoing pandemic COVID-19 outbreak has quickly spread to almost all countries in the world. As of 29 June 2021, the USA is at the top in terms of COVID-19 infections and deaths, 34,511,636 and 619,595, respectively whereas India is the second-most COVID-19-affected country with 30,316,897 confirmed cases and 397,668 deaths. Although Brazil is the third-most affected country with 18,448,402 COVID-19 cases but reported to have 514,202 deaths, which is higher than India [6,7].

The World Health Organization (WHO), in January 2020, has declared a global health emergency just a month after its first reported case in China. Though WHO estimated the fatality rate of this disease to be around 4%, older people and persons with pre-existing comorbidities such as diabetes, cancer, hypertension, kidney failure, etc. are usually at high risk of losing their lives. Accordingly, the course of infection caused by SARS-CoV-2 can be approximately categorized into three stages: stage I, incubation period in which virus may or may not be detected; stage II, non-severe symptomatic period that shows the residence of the virus in throat swab; stage III, the severe respiratory symptomatic condition with maximal viral load [8,9]. The incubation period of COVID-19 in familial clusters was found to be around 2–14 days. The clinical features of COVID-19 include fever, cough, muscle pain or lethargy and less commonly, headache, coughing up blood and diarrhea [10]. The COVID-19 infection in the lung parenchyma results in severe interstitial inflammation of the lungs, which is clearly evident from computed tomography (CT) scan images as ground-glass opacity in the lungs. This inflammation, even though initially involves only a single lobe, later can expand to multiple lobes of both lungs. The histological investigation of lung biopsy specimen collected from COVID-19 patients demonstrated diffuse alveolar damage, cellular fibromyxoid exudates, hyaline membrane formation and desquamation of pneumocytes [11]. Cytokines are intercellular messengers produced from innate immune cells and demonstrated an important role in the regulation of the immune response. Cytokine storm syndrome is the phenomenon in which the body level of proinflammatory cytokine increases due to certain drugs or microbial infections [12].

The patient's ability of resistance demonstrated an essential role in fighting against any viral infection and developing a long-lasting protective immunity. T cells play a crucial role in antiviral immunity, but their actual function in developing protective response is still under investigation. The appearance of T-cell responses right from the beginning and till the emergence of long-lasting protective memory T cells greatly relies on innate immune responses. Innate immunity involves a wide variety of cells such as antigen-presenting cells (APCs), macrophages, mast cells, monocytes and natural killer (NK) cells. An innate immune cell can precisely identify the molecular alteration induced by a viral infection; therefore, adaptive immune responses will be promptly initiated, such as phagocytosis and cytotoxicity by NK cells. Innate immune cells do also play a significant role in immune effector response followed by induction of antibody formation through Fc receptor expression [13]. The interplay between innate and adaptive immunity during viral infection constitutes a vital role and viral antigen uptake by APCs leads to cross-presentation of processed antigens, through major histocompatibility class I (MHC-I) molecules, to induce infected cell-specific CD8^+^ T cells, antibody and memory T cells for clearing infected cells and developing long-lasting protective immunity [14]. The development of an effective and stable COVID-19 vaccine primarily depends on its formulation strategy and vaccine nanocarriers such as nanoparticles, liposomes. Most of the engineered vaccines are unstable and prone to degradation during processing, storage and transport. Therefore, the development of a stable and effective vaccine requires a meticulous selection of non-immunogenic excipients and appropriate processing parameters.

Here, we reviewed the role of innate immunity in the successful treatment of COVID-19 by highlighting the mechanisms by which innate immune cells can identify invading viruses, initiate and promote adaptive immunity. Additionally, we have discussed various strategies for targeting potential immunological targets with which viral infection can be suppressed effectively. Further, we briefly discussed several attempts that have been carried out for manipulating innate immune responses during COVID-19 treatment. We have limited our discussion to currently available and ongoing clinical investigation-based immunotherapy for the effective management and prophylaxis of SARS-CoV-2 infection.

## Structural composition of coronaviruses & their function

**Figure 1.**
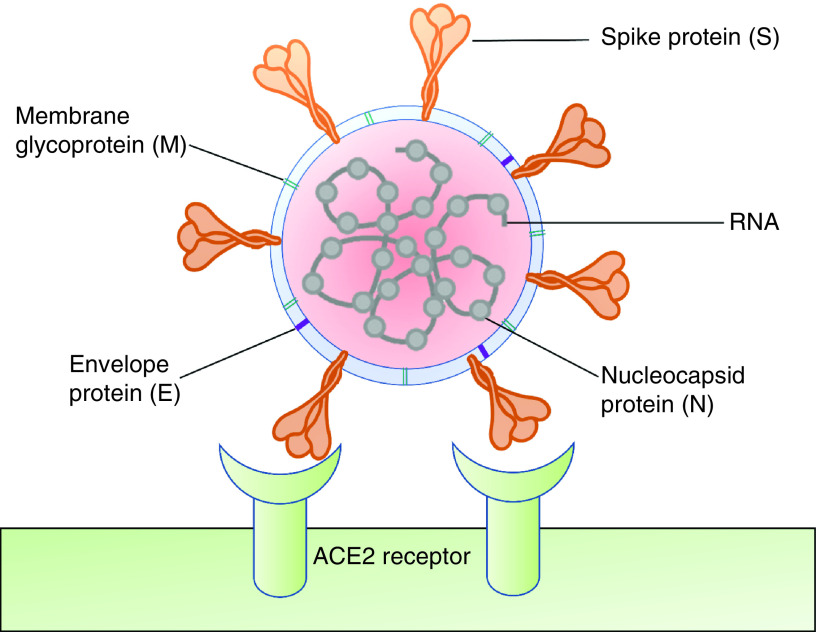
Coronavirus structure with viral ACE2 receptor on the host cells surface. ACE2: Angiotensin converting enzyme 2.

Coronaviruses consist of a family of enveloped, unsegmented and single-stranded positive-sense RNA viruses belonging to the Coronaviridae family, which can infect not only humans, mammals but also some birds. The virus which has caused this lethal pandemic, SARS-CoV-2, has a 29.891 kb long genome with 38% of G + C content and is enveloped by nucleocapsid [6,15]. The surface morphology studies from electron microscopy have revealed that SARS-CoV-2 has a virion diameter of 60–140 nm and spike length of 9–12 nm which has been correlated to the shape of a solar corona. The molecular characterization of SARS-CoV-2 has demonstrated that this new betacoronavirus belongs to the subgenus *Sarbecovirus* [16]. Coronaviruses are identified to have four vital structural proteins, namely spike (S), membrane (M), envelope (E) and nucleocapsid (N) (Figure 1). The S protein is a viral transmembrane protein that belongs to class I, having multifunctional properties. The size of this widely available S protein varies from 1160 to 1400 amino acids and the virion also consists of trimer on its surface, giving the virus a ‘corona’ or crown-like appearance. The functional property of this S protein is to help virion particles to invade the host cells.

The M protein of SARS-CoV-2 is located mainly inside the virion, which gives a concrete architecture to the viral envelope. This M protein binds to the viral-enveloping nucleocapsid which acts as the main organizer of viral assembly. The viral E protein is highly cryptic and smaller among the principal structural proteins. It demonstrated a key function in the pathogenesis, assembly and release of the virus. It is a small integral membrane polypeptide that serves as an ion channel for a virus known as viroporin. Deactivating or disappearance of this protein is associated with modified virulence of virus due to alteration in conformation and tropism. The N protein of coronavirus has a multifunctional role such as complex formation with viral genome, facilitation of M protein interaction needed during virion assembly and thus enhancing the transcription efficiency of the virus. Phylogenetic analysis suggested that SARS-CoV-2 is nearly identical to SARS-CoV with a similar way of cell entry. Further, as SARS-CoV-2 has almost 77.5% resemblance to SARS-CoV, it is anticipated that the transcriptome and immunological regulations of SARS-CoV-2 would have a high degree of overlap with SARS-CoV. Since SARS-CoV and SARS-CoV-2 have such a high degree of resemblance, many previous studies on SARS-CoV can be broadened to SARS-CoV-2 to speed up pharmaceutical and vaccine development against COVID-19. The apparent similarity between these two viruses may potentially boost the understanding of SARS-CoV-2 immunologically. As evident from currently ongoing parallel studies regarding SARS-CoV-2, vaccines may emerge soon for human users [16–21].

**Figure 2.**
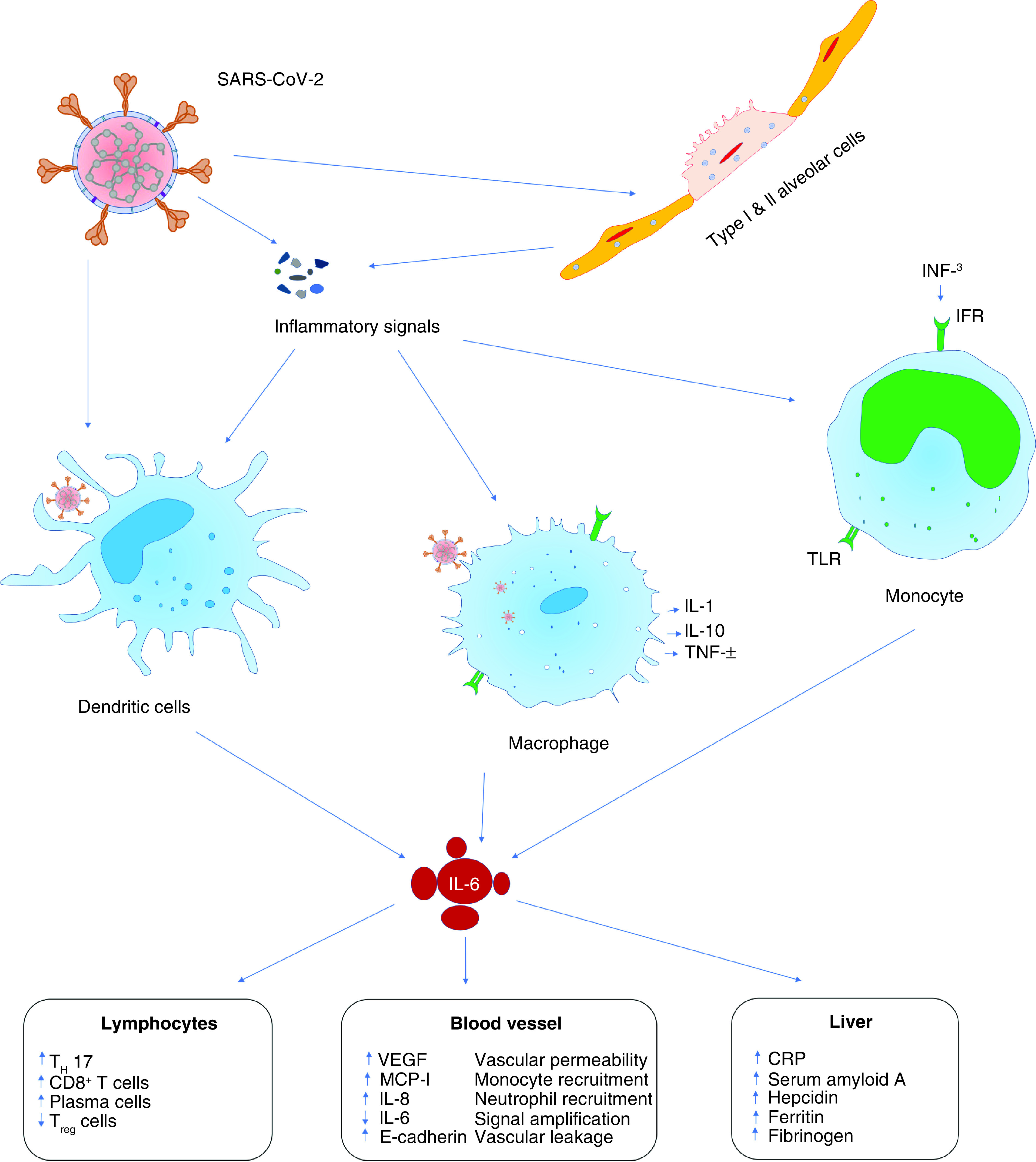
Cytokine storm induced during COVID-19. CRP: C-reactive protein; IFR: Interferon receptor; MCP-1: Monocyte chemotactic protein-1; TLR: Toll like receptor.

It is found that SARS-CoV-2 recognizes and binds to ACE2 receptor through the spike protein, which leads to rapid activation of CD4^+^ T cells and their subsequent proliferation and differentiation into T helper 1 (Th1) cells, which secrete proinflammatory cytokines such as IL-6, IFN-γ and granulocyte–macrophage colony-stimulating factor (G-MCSF). Phosphatase and tensin (PTEN) are essential in the induction of dendritic cells (DCs), B cells, T cells and release of proinflammatory cytokines and they all work together with downregulated genes in the COVID-19 patients to promote cytokine storm. SARS-CoV-2 pathogenesis has been linked to the induction of proinflammatory cytokines triggered by the stimulation of at least five different pathways, along with the nuclear factor of activated B cells (NF-B), nuclear factor of activated T-cells (NF-AT), interferon regulatory transcription factor-3 (IRF-3), interferon regulatory transcription factor-7 (IRF-7), activating transcription factor-2 (ATF-2)/jun and jun/fos (activated protein-1). Additionally, studies on the reactome pathway suggested that PTEN homolog demonstrated a pivotal role in SARS-CoV-2 infection by activating DCs, B-cells, T-cells and release of proinflammatory cytokines that includes IFNs, tumor necrosis factor-α (TNF-α), IL-10, IL-4 and G-MCSF [21]. Out of these proinflammatory cytokines, G-MCSF may also trigger monocytes for further production of IL-6 (Figure 2) and other factors leading to the development of cytokine storm. Thus, patients with cytokine storms may develop acute respiratory distress syndrome (ARDS) and severe pneumonia may even lead to death [11].

## Viral antigen detection & promotion of innate immunity

When host cells detect the viral invasion, they elicit the immune response against the invading virus. The viral antigen will be initially recognized by the innate immune system, which usually identifies viral components through pattern-recognition receptors (PRRs). As the immune system detects pathogens, its corresponding response solely comprises two types of reactions: an innate immune response and an antigen-specific adaptive immune response [22]. The innate response has demonstrated a vital role in the first line of defense mechanism characterized by the rapid appearance of various cells that involve in first-line defense such as neutrophils, monocytes, macrophages, eosinophils, mast cells, DCs, etc. These cells detect the pathogen-associated molecular patterns (PAMPs) with the help of pattern-recognition receptors such as toll-like receptors (TLRs), which are widely present on APCs. PAMPs are conserved in microorganisms that can infect humans. APCs such as DCs do amplify the inflammatory responses and activate cellular immunity through antigenic presentation [23]. Viral replication will be restricted by activated innate immune cells through the activation of intricate signaling networks. Following, downstream effector mechanisms exert a major role in triggering innate immune responses and initiation and promotion of adaptive immunity. Innate immunity is thus quite crucial for the initial recognition of viral invasions and subsequent activation of adaptive immune responses. The recognition of viral components such as single- and double-stranded RNA and single-stranded DNA involves three different receptors such as retinoic acid-inducible gene I (RIG-I)-like receptors (RLRs), TLRs and NOD-like receptors (NLRs). RLRs and TLRs are found to play an essential role in the generation of type I interferons and pro-inflammatory cytokines in a cell-specific manner. While the RLRs are pivotal in the detection of ribonucleic acid (RNA) viruses in different cells and by utilization of TLRs, DCs identify viral invasion in the host. On the other hand, NLRs demonstrated a major role in the generation of maturation interleukin-1β through activation of caspase-1 and double-strand RNA (dsRNA) stimulation [24–26].

### Innate immune response during SARS-CoV-2 infection

**Figure 3.**
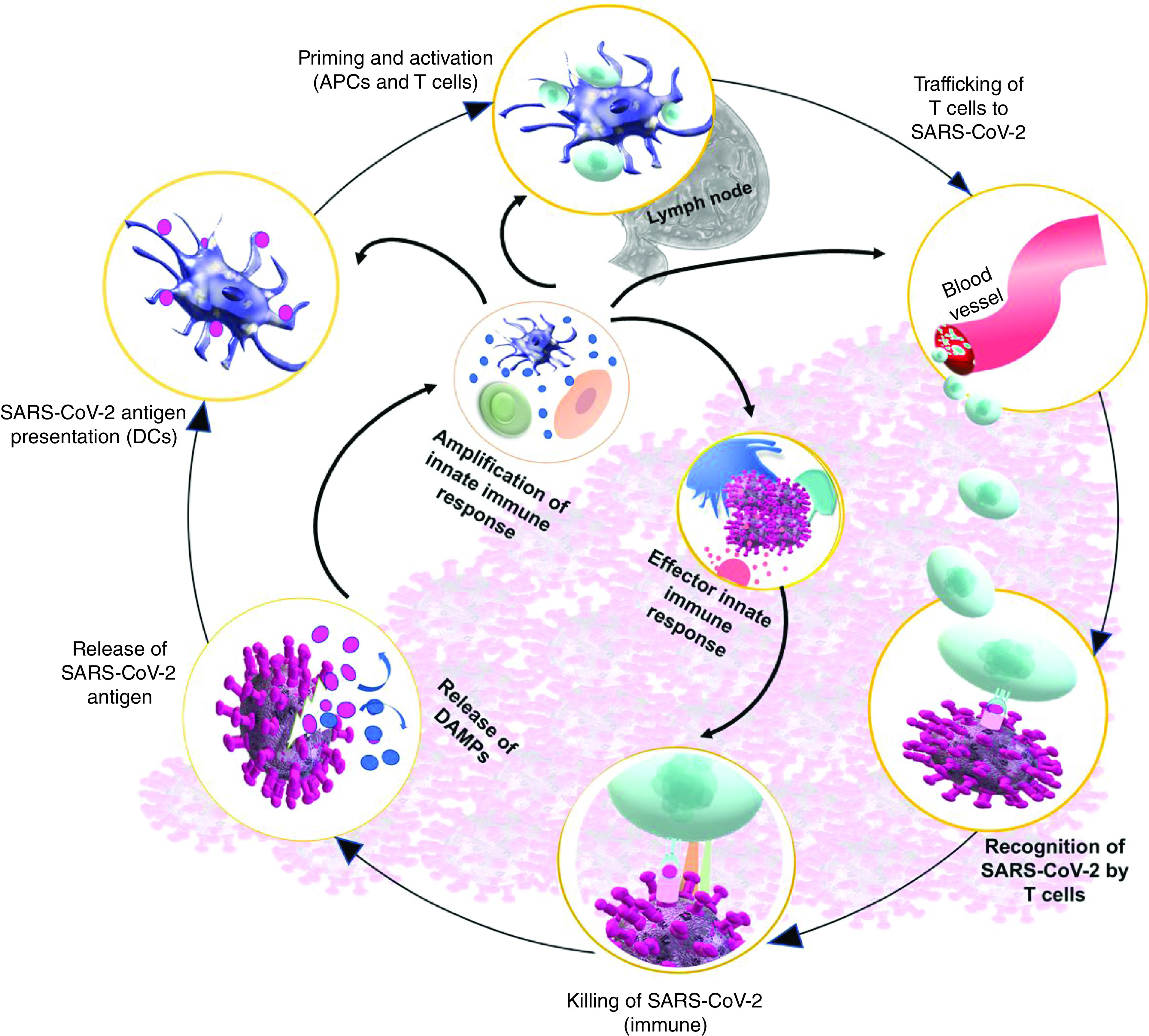
Innate immune response during COVID-19; detection by dendritic cells and killing by cytotoxic T cells. APC: Antigen presenting cell; DAMP: Damage-associated molecular pattern protein; DC: Dendritic cell.

The activity of innate immunity largely depends on the involvement of a wide variety of cells that include APCs such as DCs, white blood cells, mast cells, lymphoid cells and NK cells [27,28]. T cells are vital in fighting against viral infection through immune responses and are capable of neutralizing the invading viruses with the help of CD8^+^ cytotoxic T cells (Figure 3). These cytotoxic cells can release a range of molecules such as perforin, granzymes and IFN-γ, which selectively eliminate viral components from the host. T helper cells (CD^+^4), on the other hand, simultaneously assist CD^+^8 cells and B cells for further enhancing their capability to clear pathogenic viral components [29–31]. However, prolonged immune stimulation by invading viruses may lead to T-cell exhaustion, resulting in diminished cytokine-producing ability and reduced activities. Since an effective immune response is crucial for the successful recovery of COVID-19 patients, promoting the number of functional T cells in patients suffering from SARS-CoV-2 infection can be fruitful [32,33]. Recently, a study on COVID-19 patients reported that 82.1% of patients had a lowered circulatory lymphocyte counts. However, the factor that contributes to the low level of circulatory lymphocytes and their activation is yet to be discovered. The retrospective investigation from the clinical data of COVID-19 patients and comparison of the upregulation of exhaustion markers, programmed death-1 (PD-1) and T-cell Ig-mucin domain-3 (Tim-3) on the surface of CD4^+^ and CD8^+^ T cells between infected patients and healthy control subjects suggested that timely intervention is essential for patients with reduced T lymphocyte counts [13,34].

IFN-based COVID-19 immunotherapy can be a viable option for patients with multiple sclerosis who have mild-to-moderate SARS-CoV-2 infection. The expression of IFNs at the early stage of SARS-CoV-2 infection is highly correlated with the innate immune response to SARS-CoV-2. IFNs are critical in interfering with viral replication and IFN-β, in combination with lopinavir and ritonavir, has demonstrated its efficacy in the treatment of MERS-CoV. With COVID-19, early intervention of IFN-β in combination with lopinavir and ritonavir has elicited significant improvement and prevented the progression of infection to the severe stages. Thus, IFN could be paramount in the successful treatment of COVID-19 patients with mild-to-moderate symptoms [35].

## Potential immunological target for COVID-19

From the beginning of this decade, the world has been suffering from the pandemic spread of SARS-CoV-2. So, it is imperative to develop a thorough understanding of this dreadful viral infection and prevent its further spread. Finding a potential immunological target is thus essential for developing a vaccine against SARS-CoV-2. Vaccine development can be accelerated by correlating the genetic identity among SARS-CoV-2 and well-studied SARS-CoV, whose outbreak was in the year 2003. A group of B-cell and T cell-specific epitopes were isolated from spike (S) and nucleocapsid (N) proteins that mapped analogously to SARS-CoV-2 proteins and were established by evaluating experimentally identified SARS-CoV-derived B-cell and T-cell epitopes in its immunogenic structural proteins. Currently, the mutation was not seen in the above-mentioned epitopes among the 120 identified SARS-CoV-2 sequences; developing vaccines by immunological targeting of these epitopes may thus essentially protect from COVID-19 [36–39].

### Targeting viral spike (S) protein

Viral spike (S) protein is vital in gaining entry into the human cell during SARS-CoV-2 infection [40]. It is observed that the binding of viral S protein to the angiotensin-converting enzyme 2 (ACE2) receptor of the hosT-cell promoted the viral entry into the cells. It is also noted that the novel coronavirus identifies ACE2 more readily than SARS-CoV. Thus, developing a strategy for inhibiting the interaction between viral S protein and ACE2 can be a beneficial approach [41]. In fact, the S protein receptor-binding domain (RBD) is an essential target for those virus-neutralizing antibodies. Since the SARS-CoV-2 S protein showed high similarity to that of SARS-CoV, the currently accessible SARS-CoV neutralizing antibody (CR3022) was reported to adhere efficiently with SARS-CoV-2 RBD [42,43]. Nevertheless, Zheng *et al.* earlier revealed that at least 85 % of RBD-antibody epitopes in SARS-CoV-2 had revealed considerable changes compared with SARS-CoV, suggesting the dire need for the development of new SARS-CoV-2 specific mAbs [43]. Notably, a randomized, regulated, pilot clinical trial is in the development phase to further determine the impact of recombinant human ACE2 (rhACE2; GSK2586881) in subjects with severe COVID-19 (NCT04287686). S protein-derived cellular invasion has indicated that it relied not only on ACE2 but also on the targeted cellular transmembrane serine protease 2 (TMPRSS2) for invasion [44]. Camostat mesylate is a clinically approved drug, which inhibits TMPRSS2 leading to a significant reduction of COVID-19 infection in the lung cell lines [45]. Also, the fusion of the virus with the patient's cell membrane is promoted by the heptad repeat 1 (HR1) and heptad repeat 2 (HR2) of the novel coronaviruses. Xia *et al.* realized that HR2-derived peptides (HR2P) and EK1 (a modified OC43-HR2P peptide) efficiently inhibited the fusion of novel coronavirus with the cell membrane [46]. Furthermore, an *in vitro* study demonstrated that hydroxychloroquine, a clinically approved antimalarial drug, was found to be effective against COVID-19. Currently, numerous clinical trials are underway to establish the safety and efficacy profile of hydroxychloroquine for COVID-19 treatment. The proposed mechanism behind its effectiveness in COVID-19 could be the impairment of endosome-mediated viral entry [47–51]. Thus, targeting the viral S protein and its associated mechanisms may lead to the prevention of viral entry into the hosT-cell and protects from COVID-19 infection.

### Targeting SARS-CoV-2 replication cycle

Numerous antiviral therapeutics are being designed for targeting viral proteases, polymerases and methyltransferases in an attempt to restrict viral replication. A lot of antiviral drugs are under clinical trials, such as remdesivir, favipiravir, lopinavir/ritonavir and arbidol for establishing their therapeutic safety and efficacy in COVID-19 treatment [52]. In a study, remdesivir is found to have promising therapeutic outcomes in COVID-19 treatment. It is a prodrug of adenosine analog whose functional segment can be incorporated into viral RNA with the help of RNA-dependent RNA polymerases, leading to inhibition of RNA synthesis. Wang *et al.* have reported in an *in vitro* study that remdesivir can efficiently prevent the growth of novel coronavirus [53]. Intravenous injection of remdesivir has adequately improved the clinical condition of first reported patients in USA [54]. Similarly, guanine analog-based antiviral drugs such as favipiravir and ribavirin are approved for the treatment of various viral infections [55]. However, a further clinical investigation is a prerequisite to establishing their activity against COVID-19. Viral protease inhibitors such as lopinavir and ritonavir can target SARS-CoV-2 major proteinase known as 3C-like protease (3CLpro). This proteinase is capable of generating the polypeptide translation product from the genomic RNA into the target proteins [56]. Ribavirin is an antiviral drug that acts by inhibiting viral RNA-dependent RNA polymerase. An i*n vitro* study for the treatment of SARS-CoV has demonstrated that a higher dose of ribavirin (1.2–2.4 g) must be administered orally every 8 h to sufficiently inhibit the viral replication. The clinical trial also suggested that ribavirin is ineffective in treating SARS-CoV-2 and is found to cause hematological and hepatic toxicities [35]. Targeting various enzymes and intermediates, which have a vital role in viral replication, may be another viable alternative for developing novel therapeutics that can restrict viral progression.

### Targeting the complement system during COVID-19 immunotherapy

**Figure 4.**
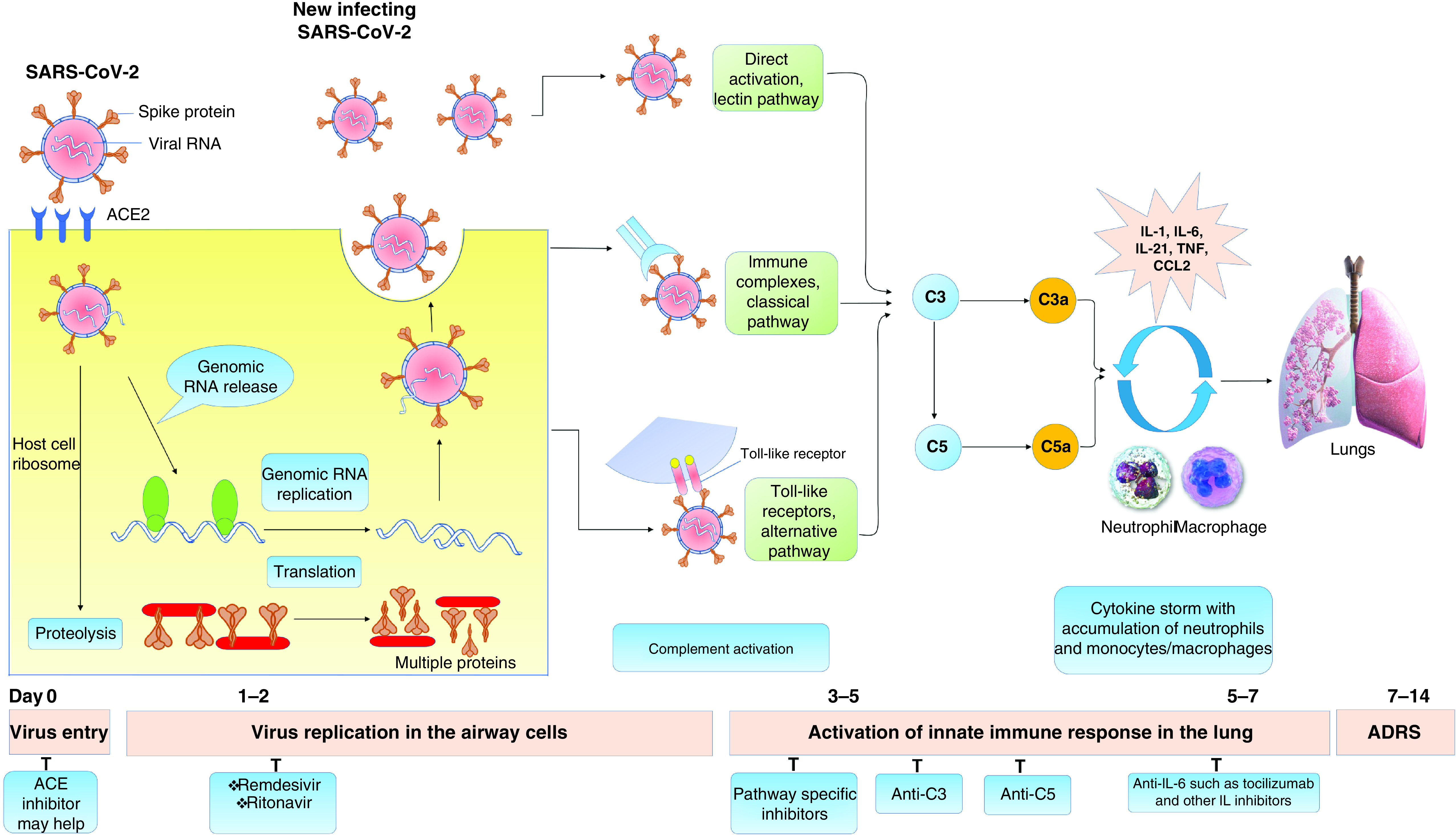
Targeting complement system for COVID-19 treatment. ACE: Angiotensin converting enzyme; ADRS: Acute distress respiratory syndrome; CCL2: Chemokine ligand 2.

The complement system is a critical component of the immune system and also acts as a stimulus for numerous pro-inflammatory symptoms in the host as shown the Figure 4 [57]. Gralinski *et al.* observed that the complement 3 (C3) activation intensified the coronavirus disease-based ARDS. Considering the comparable viral load in the lungs, a study reported that C3-insufficient mice, when infected with coronavirus, had a less severe respiratory impairment which was well correlated with lower neutrophil and inflammatory monocyte infiltration and reduced cytokine and chemokine levels in either of the lungs and the sera [58]. The above investigation indicated that inhibition of C3 could also alleviate the problems of the inflammation in the lungs induced by SARS-CoV-2 (Figure 4). This substantial reduction in pulmonary neutrophil invasion and the decreased intrapulmonary IL-6, as well as their plasma levels, are also seen in SARS-CoV. The study on C3-insufficient mice has demonstrated that C3 inhibitors can be combined with anti-IL-6 regimes for superior clinical outcomes [59].

Besides, the upstream location of C3 signals in the innate immunity is also asserted for the superior anti-inflammatory effect of the C3-blocking in COVID-19 patients with AMY-101 (a potent C3 inhibitor drug). C3 inhibition may concurrently block the production of C3a and C5a and also prevents intrapulmonary activation of C3. This, in turn, blocks the release of IL-6 from bronchial macrophages and from such cells that express C3a receptors (C3aRs) and/or C5a receptors (C5aRs), hence lowering the lung inflammation. This study has also established that IL-6 release was hindered through an *ex vivo* whole blood sample that employs AMY-101 with compstatin C3 inhibitors [60,61]. A recently published research has suggested a widespread complementary activation of C3a generation and C3 fraction deposition in patients with severe COVID-19 infection. There was also a significant increase in serum C5a. Pertinently, the management of patients with anti-C5a antibodies has contributed to an augmented clinical benefit by simultaneously rising lung oxygenation and reducing systemic inflammation. For nearly 15 years, the C5 inhibitors have been safely used in hospitals and the well-known function of C5a–C5aR in ARDS pathophysiology is indeed confirmed by their use in initial clinical trials.

However, the effect of the C5 inhibitors can be partial, which may skew their efficacy in the residual terminal pathway during severe complement activation in COVID-19. Additionally, C5 inhibitors do not impact the C3a-C3a receptor axis [62,63]. Proximal complement inhibitors that primarily target the C3 complement system may be potential candidates for COVID-19, although these are still in clinical research and none of them have been approved and only minimal evidence is available from Phase II clinical trials. Provided the complement system's structure, both of these agents may theoretically prevent initial steps that trigger lung inflammation [64].

The potential benefits of targeted complement inhibition through both alternative and classical pathways are still to be established. Similarly, the summation of clinical features from ARDS development with established inflammation biomarkers (plasma IL-6 concentrations, ferritin and C-reactive protein) will make patients detectable and thereby benefited from complement inhibition. In the severe SARS-CoV-2 infection, inhibition of C3 has the power to manage not only ARDS but also vascular inflammation within the liver beds, heart and other vital organs, which otherwise tends to be risky in extreme conditions. Complementation is a significant defensive mechanism against infectious microbes, but their hyper-activation and over-regulation can lead to collateral injury to tissues. Complement system inhibitor-dedicated pharmaceutical industry and scientists should come forward in these difficult moments of COVID-19 and bring more drug candidates into clinical application [65,66].

### Targeting TH17 immune responses

T helper 17 cells (Th17) are pro-inflammatory T helper cells and they have been named so because of their ability to produce IL-17. Th17 cells have demonstrated a vital role in maintaining barriers on the mucosal layer for neutralizing and clearing invading pathogens. Treg17 cells constitute a subpopulation of Th17 cells that exercise protective activity against pathogens. They are also reported to be crucial in mediating innate responses and further developing adaptive immunity so as to protect the host from pathogenic infections [67]. In the initial stages, cytokines released from Th17 cells seem to be vital in developing antimicrobial immunity evidently due to their part in triggering the activation and migration of neutrophils at the targeted site. It has been found that Th17 is also crucial in promoting the synthesis of antimicrobial and activating enterocytes proliferation. Thus, Th17 has a vital role in protecting the body from extracellular pathogenic microbes [68].

Clinically, numerous COVID-19 patients have been reported as having developed ARDS, which trigger severe damage to vital organs such as lungs, liver, kidney and heart. This damage to vital organs is the consequence of the cytokine storm, causing higher levels of various interleukins to be released. Patients with severe COVID-19 infection were reported to have even higher levels of inflammatory IL-2, IL-6, IL-7, IL-10. Out of these, many interleukins are involved in the immune responses mediated by TH17, which promote vascular permeability, leakage, neutrophil recruitment at the site of inflammation leading to tissue damage. It has been observed that IL-22 contributed to the development of life-threatening edema in patients infected with SARS-CoV-2 [69–71]. Xu *et al.* reported that patients with severe COVID-19 have an abnormally elevated number of TH17 cells in peripheral blood, an indication of cytokine storm. Thus, a higher number of TH17 cells produce a surge in the levels of IL-17, which further worsened the clinical condition of COVID-19 patients due to IL-17-provoked acute lung injury [10]. A similar kind of cytokine storm was also reported in the MERS-CoV, SARS-CoV and H1N1 influenza pandemics. Cytokine storm produced by TH17 cell response has resulted in the generation of pulmonary edema; hence targeting the TH17 may be significant for COVID-19 patients with aggressive TH17 immune profiles. Since it may require a long time and rigorous research to develop specific drugs or vaccines for COVID-19 treatment, repurposing the currently available medications for the treatment of SARS-CoV-2 could be a great opportunity [72]. Also, numerous antibodies are available that can inhibit IL-17-mediated cytokine storm, but all these antibodies are expensive and have a narrow efficacy spectrum. Several anti-IL17 mAbs are in clinical trials that may specifically prevent lung injury mediated by these cytokine storms.

Additionally, Fedratinib, a Janus kinase-2 inhibitor (JAK2 inhibitor), can prevent the generation of TH17-mediated cytokine storm; therefore may improve the clinical condition of severely affected COVID-19 patients. Fedratinib can be prescribed in combination with other antiviral drugs. Since inhibition mediated by fedratinib is reversible, short-term therapy with this JAK2 inhibitor before progression of the illness from serious to the critical stage would not affect the TH17 responses required for innate immunity and antiviral immunity against invading viruses [73,74].

## Antibody-based COVID-19 treatment

Monoclonal antibodies (mAbs) based therapy could be the most useful treatment strategy for individuals infected with SARS-CoV-2. Patients recovering from this disease have demonstrated potentially neutralizing antibodies against this COVID-19 infection in their blood samples [4]. Previous studies on MERS-CoV reported that a group of mAbs are actively targeting the specific domains of the MERS-CoV S protein. These domains include six distinct epitope groups, which involve in receptor binding, membrane fusion and sialic acid-binding sites that comprise vital entry functions of S protein [75]. Further research has supported the fact that passive immunization with potentially neutralizing mAbs provided significant protection in mice with MERS-CoV. Such antibodies may be promising in promoting the humoral immunity for protection against the developing coronavirus by considering vital epitopes and functions of the S protein [76]. The cross-neutralization ability of SARS-CoV and the antibodies considerably relies on the resemblance of their RBD; therefore, SARS-CoV RBD-specific antibodies could cross-neutralize SARS-like (SL) CoVs, in other words, bat-SL-CoV strain WIV1 (RBD with a difference of eight amino acids to that of SARS-CoV), but not bat-SL-CoV strain SHC014 (24 amino acids difference). With the help of analogous analysis of COVID-19 RBD with that of SARS-CoV, preferred mAbs specific to RBD can be found, which can cross-neutralize SARS-CoV-2 [77]. Shah *et al.* found that Lys417 mutation in cRBD resulted in better electrostatic interaction with ACE2 that may help cells to recognize receptors more quickly. At the cRBD-ACE2 interface, electrostatic and hydrophobic colligation reinforced this interaction. Additionally, they observed that Val-to-Lys417 mutation in RBD of SARS-CoV-2 led to the establishment of enhanced electrostatic attraction with ACE2-binding. SARS-CoV-2 neutralization by anti-SARS-CoV mAbs may be hampered by Pro-to-Ala475 substitution and Gly482 insertion in the AGSTPCNGV-loop of RBD [78].

### Human mAbs CR3022

The US-based Biotech firm, Regeneron, is trying to identify potent and specific mAbs for combating COVID-19. A perfect therapeutic intervention recommended for COVID-19 treatment was the combination therapy that consists of mAbs and the drug remdesivir. A human mAb CR3022, specific to SARS-CoV, is known to bind with SARS-CoV-2 RBD, insisting its potential as a therapeutic agent for COVID-19 treatment. Further, it was found that other SARS-CoV-specific neutralizing mAbs such as m396 and CR3014 failed to bind with the S protein of SARS-CoV-2, suggesting that a particular level of similarity is essential for the cross-neutralization activity between the RBDs of SARS-CoV and SARS-CoV-2 [42,79]. Although many attempts were made to find antibodies that can prevent infection, antibodies' potential to combat infection via Fc interactions may be crucial in eradicating the virus. In a study, Atyeo *et al.* noted that mAbs CR3022 was able to cross-neutralize viral infection. They performed Fc engineering for mapping the therapeutic effectiveness of the Fc. Apparently, the Fc-enhanced mAbs CR3022 provided some viral control in mice, but it was also associated with substantial morbidity in both mice and hamsters, possibly as a result of eliciting an inflammatory reaction [80]. Further investigation is essential before establishing the efficacy of such combination therapy.

### Tocilizumab recombinant humanized mAbs

Tocilizumab is a recombinant humanized anti-human IL-6 receptor mAb, currently in use for the treatment of rheumatoid arthritis [81,82]. Tocilizumab preferably binds with IL-6 receptor and inhibits signal transduction. Long-term clinical studies suggested that tocilizumab is well tolerated and safer for human use. A recent study has revealed that higher levels of proinflammatory cytokines such as IL-2, IL-6, IL-7, IL-10 and IFN-γ were observed in the patients severely infected with SARS-CoV-2. A clinical study also demonstrated that COVID-19 symptoms such as hypoxemia and CT opacity changes were alleviated immediately after the intervention with tocilizumab in a group of patients, indicating that tocilizumab could be an efficient therapeutic approach for COVID-19 treatment. However, in few patients, the clinical symptoms got worsen over time such as chest stiffness, difficulty in breathing and even respiratory failure [83]. A recent study confirmed that a decline in the lymphocyte content is the potential biomarker for diagnosis and judging the severity of COVID-19. The study also reported that minimal lymphocytic content was found in 85.0% of patients, whereas 52.6% of patients returned to normal after tocilizumab intervention. Recently, tocilizumab has been approved in China for treating COVID-19 patients with pneumonia and an increased level of IL-6 based on a randomized multicentered clinical trial (ChiCTR2000029765) [84]. Some of the studies suggested that viral mutations do not alter the efficacy of the tocilizumab [85]. Therefore, tocilizumab can be a promising treatment strategy for COVID-19 patients associated with febrile and inflammatory storm responses. However, multicentered clinical studies in large population groups are essential for establishing the efficacy of tocilizumab for the treatment of COVID-19 throughout the globe.

### Antibodies that specifically neutralize SARS-CoV-2

Enhancing humoral immunity through antibodies can be highly beneficial in the neutralization and prevention of viral infections [86]. Therefore, the design and production of the relevant surface epitope-targeted neutralizing mAbs is a less explored but highly viable option for targeting SARS-CoV-2 [65]. AbCellera (Canada) and Eli Lilly and Company (USA) are designing active mAbs in collaboration that could neutralize viral components in COVID-19 patients. For this, they performed the screening of more than five million immune cells obtained from the first recovered COVID-19 patients in USA. They have also detected more than 500 COVID-19 antibody sequences; their screening is still underway to select the best one. As evident, such a strategy may take a long time and requires vast resources, so simultaneous manufacturing may be propitious to supply and meet the ever-increasing demand for these neutralizing antibodies. Similarly, many pharmaceutical and biotechnology-based companies such as Vir Biotechnology, Inc. (CA, USA), ImmunoPrecise (VC, Canada), Mount Sinai Health System (NY, USA) and Harbour BioMed (HBM; Hong Kong, China) are also in the process of developing the best antibody that can efficiently neutralize the COVID-19 viral infection [87]. As of now, all of the above-mentioned antibodies are under preclinical stages and the best one is assumed to have both protective and treatment effects [88].

#### Effect of mutations in binding efficiency of mAb to RBD

The development of SARS-CoV-2 mutations over time can significantly impact the efficiency of the vaccines and S protein-directed neutralizing monoclonal antibodies (nAbs). The neutralization of SARS-CoV-2 S protein, as a technique of post-exposure prophylaxis, has been attempted in one of the three different ways: biologically resembling ACE-2 to interact with the virus's RBD and prevent the ACE-2-RBD complex, coupling to RBD without resembling ACE-2, and complexing RBD but without blocking ACE-2-RBD interaction. Furthermore, SARS-CoV-2 evolution via hypermutation occurrences may hinder nAb identification and thus neutralization capability [89].

Research has revealed numerous major epitopes in the RBD that were linked to existing strains and had an impact on receptor binding efficiency. The E484K mutation (found in the S.501Y.V2 and B.1.1.28 lineages, respectively, in South Africa and Brazil) showed an enhanced affinity toward the host ACE-2 receptor. They also revealed significant variability in the implications of mutations across people and within the same person over time. Mutations in the spike protein occurred as a result of positive selection in the vicinity of convalescent serum antibodies, enabling a substantial drop in neutralization capability and subsequent interaction with the ACE-2 receptor. SARS-CoV-2 was found to be neutralized by this convalescent plasma at first, but over time, numerous mutations provided resistance to the virus from neutralization [90].

Further, a blend of two nAbs like BRII-196/BRII-198 and REGN-COV2 is said to stop viruses from escaping by binding to two different neutralizing epitopes. In such a case, two concurrent viral mutations at two different genetic locations would be required for a viral escape to occur [89].

### Anti-C5a monoclonal antibody

As evident from many ongoing studies, the activated complement system has largely contributed to acute lung injury. Notably, C5a is the activated complement system generated by cleavage of C5 and is potentially responsible for tissue injury. The activated complement system promotes the migration of neutrophils and T-lymphocytes at the cellular site and enhances the pulmonary vascular permeability. Clinical studies have suggested that anti-C5a therapy can minimize lung injury by reducing vascular permeability and inhibiting the migration of neutrophils into the alveolar space [91]. Thus, there are some anti-C5a mAbs such as BDB-1, IFX-1 and InflaRx designed by Beijing Defengrei Biotechnology Co. (Beijing, China) and Beijing Staidson Biopharma (Beijing, China), respectively, for targeting inflammatory responses elicited by the activated complement system. Hence, these anti-C5a mAbs are expected to reduce the lung injury produced by COVID-19 [92].

## Convalescent plasma-based COVID-19 immunotherapy

Convalescent plasma is the plasma that contains the antibodies or immunoglobulins against those viral infections from which the patient is successfully recovered. Convalescent plasma was used as a last attempt to increase the survival rate of SARS patients whose health was continued to worsen following pulsed methylprednisolone therapy [93]. Moreover, several clinical investigations reported that patients who underwent convalescent plasma therapy had a minimal hospital stay and lower death rate than those individuals who were not received convalescent plasma therapy. In 2014, WHO recommended the use of convalescent plasma, obtained from the patient who completely got cured of the Ebola virus infection, as empirical treatment of the same outbreak. Similarly, in 2015, a protocol of convalescent plasma therapy for the treatment of MERS-CoV was established [94].

In 2009, Hung *et al.* demonstrated in a prospective cohort study that convalescent plasma therapy for the H1N1 influenza pandemic has considerably abated the relative mortality risk. Furthermore, subgroup analysis suggested that following convalescent plasma therapy, viral load was substantially lowered on days 3, 5 and 7 when supported with intensive care facilities [95]. Hung *et al.* in a multicentered, double-blind, randomized controlled clinical trial, reported that using convalescent plasma therapy in subjects with severe H1N1 influenza virus infection has resulted in a minimal viral load and minimized mortality within 5 days of onset of symptoms [96]. In another study, Mair-Jenkins *et al.* demonstrated that a considerable decline in the mortality rate of patients with SARS was observed after multiple convalescent plasma dosing, without any adverse events. One potential reason for the success of convalescent plasma therapy is that viremia can be suppressed by the antibodies from convalescent plasma [97]. Recently, Schoofs *et al.* demonstrated that a patient's humoral immunity against HIV-1 could be enhanced through 3BNC117-mediated immunotherapy (broadly neutralizing antibody) [98].

An *in vivo* clinical study also revealed that these antibodies' merits were not only restricted to nascent viral destruction and preventing new infection but also speeding up clearance of infected cells. In numerous viral infections, peak viraemia levels can be found in the first week of viral infection. The patient typically elicits a primary immune response by days 10–14, which is followed by viral clearance. Therefore theoretically, administering the convalescent plasma at the earlier stage of the infection could be more useful [93,99]. Nevertheless, other therapies including antiviral medications, antibiotics and systemic immunoglobulins may have a direct impact on the relationship between convalescent plasma and antibody concentration. As per the WHO guidelines on COVID-19, management primarily focuses on the prevention of infection, identification and tracking of cases, and supporting treatment. Currently, there is a scarcity of specific anti-SARS-CoV-2 therapy as many of the leads are under clinical trials. Evidence indicates that convalescent plasma from the individuals who have been healed from coronavirus infections can be used as a therapy without any significant adverse effects [100]. In some of the COIVID-19 cases, convalescent plasma has been used for the treatment of SARS-CoV-2 infection. A study reported that five patients suffering from SARS-CoV-2 infection along with ARDS, treated with convalescent plasma and clinical outcomes were correlated. The results demonstrated that patient's symptoms were alleviated and got recovered soon from infection after convalescent plasma transfusion [101]. Duan *et al.* performed convalescent plasma therapy in ten adult patients with severe COVID-19. It was noted that 200 ml of convalescent plasma was well tolerated by the patients and neutralizing antibodies were able to maintain the desired concentration. This treatment strategy was able to reduce viral load within 7 days and clinical symptoms were improved efficiently within 3 days [35,102]. However, a multicentered, large-scale convalescent plasma therapy trial must be conducted to establish its safety and efficacy.

## Stem cell-based COVID-19 immunotherapy

The development of novel antiviral agents could be another choice for potentially combating the COVID-19 pandemic. But these developments are still a far way to go in order to become a reality. Additionally, stem cells based COVID-19 therapy may be an advanced and useful way of developing such antiviral treatment. Currently, numerous clinical trials, based on stem cells for COVID-19 immunotherapy, are underway to establish the safety and efficacy of immunotherapy. Many of us may have a query, why and how stem cells can fight against such deadly coronavirus. Stem cell transplantation induces aging in the host and; T cells are identified to express the marker for aging after bone marrow stem cell transplantation [103]. Therefore, stem cell transplantation may compromise immunity which is essential for fighting against coronavirus. This phenomenon is not found to occur when molecules from stem cells are used instead of whole stem cells. The rationale behind this therapy is as follows; as part of innate immunity, stem cells are found to produce antimicrobial peptides (AMP) that are essential to fight against viral infection. Stem cell activation led by viral infections triggers the production of AMP, which is important for stem cell protection and viral combat. It is also reported that stem cells are found to be contained in a compartment and protected from viral infection, even though the outer compartment has a heavy viral load. This all happens due to the AMP released from stem cells. Recently, many studies demonstrated that stem cells had displayed a vital role in mediating adaptive immune responses. Stem cells are also found to have a memory that plays a major role in protecting the body against such viral infections in the future. Further, a DNA segment encodes the activation center for inflammasome, a unique protein conglomerate that contributes to the skin defense mechanism against bacterial and viral infections [104].

Wu *et al.* reported that stem cells are biologically programmed to upregulate antiviral interferon-stimulated genes (ISGs) that boost the host immunity to defeat infections led by the viral attack. Also, β-glucan, a portion of the bacterial and fungal cell wall, stimulated IL-1β secretion, which is responsible for instructing both hematopoietic stem cells (HSCs) and myeloid progenitors. Particularly in comparison with immature HSCs, these instructed HSCs and myeloid progenitors are capable of fighting inflammatory obstacles effectively. Further, IL-1β-instructed HSCs displayed significant modification in their metabolic energy, showing increased glycolysis and biosynthesis of cholesterol, adaptations that began to be crucial to impart downstream functional alterations in the β-glucan-dependent HSC instruction pathway. With the integration of this stem cell training along with doctrinal adaptive immune cell conditioning, T-cells boost through a fiber-rich supplement that stimulates an allostatic and is pro-resolving for viral diseases [105]. Thus, the development of stem cell-based immunotherapy for COVID-19 infection could be new hope for humans in the battle of this deadly new coronavirus.

## Vaccines

Vaccines are the biological substances that consist of artificially processed attenuated/inactivated pathogens for inducing the body's defense mechanism to produce a specific immune response. Currently, the COVID-19 vaccines are under development that includes DNA vaccines, RNA vaccines and traditional live virus vaccines (Table 1). The S protein is identified as one of the most critical targets for designing these vaccines [106–110]. Recently, Daniel and colleges demonstrated the cryo-electron microscopic structure of a novel coronavirus S trimer that can potentially gear up the vaccine's developmental speed [111]. Additionally, Lucchese *et al.* reported the pentapeptide structure of S protein, which is unique to SARS-CoV-2. This investigation was performed by comparison of the viral and the human proteomes and it is also observed that 107 human-foreign pentapeptides were engraved in S protein. Furthermore, the pentapeptide embedded in the S protein is identified to have 66 epitope regions for efficient vaccine development. Research on the epitopes of SARS-CoV-2 suggested that T lymphocytes and B lymphocytes can also provide theoretical support for safer vaccine development [112]. Currently, more than 141 potential vaccine candidates that may have a promising outcome for COVID-19 are being tested around the world, out of which 119 vaccine candidates are being evaluated in humans. Around 50 vaccines are in Phase I clinical trials being assessed for safety and dosage, 37 vaccines are in expanded safety Phase II clinical trials, 32 vaccines are in Phase III clinical trials for large-scale efficacy evaluations [113,114]. There are nine vaccines that were approved for early or limited use, eight vaccines for full use and five vaccines are abandoned after trials. First, A Chinese CanSino Biologics developed an adenovirus-based COVID-19 vaccine called Ad5 which has demonstrated promising clinical outcomes in Phase I and Phase II clinical trials by producing a strong immune response against SARS-CoV-2. This vaccine is approved by the Chinese military and simultaneously evaluated for large-scale efficacy (Phase III) in many countries and recently, the Saudi health ministry has given approval for conducting Phase III trials in Saudi Arabia. Second, Gamaleya Research Institute has developed the Gam-Covid-Vac Lyo vaccine for COVID-19, which is based on two adenoviruses Ad5 and Ad26. Russian healthcare regulating agencies have approved the vaccine for limited use. Pfizer and the German company BioNTech developed the COVID-19 vaccine with an efficacy of 95%, which has been approved in several countries. Similarly, Boston-based company Moderna developed a vaccine against covid-19 with an efficacy of 94.5% and has been fully approved by FDA for usage in Switzerland and other countries. Another COVID-19 vaccine, Covishield, was developed by AstraZeneca in collaboration with the University of Oxford and has been approved in India and made by the Serum Institute of India [113].

**Table 1.** Current status of some potential COVID-19 immunotherapeutics.

Name of platform	Nature of therapy	Therapeutic target	Mechanism of action	Current status	Ref.
Tocilizumab	mAbs	IL-6R	mAbs specifically binds with IL-6R and prevent signal transduction. Thus, prevent cytokine storm induce lung injury	Its clinical trial results suggested that tocilizumab effective up to some extent with some serious adverse drug events	[84]
Thymosin	Hormonal therapy	T cells	Polypeptide hormone for the maturation of T cells, to be given in combination with antiviral drugs such as ritonavir, lopinavir	Currently, under clinical trial, the preliminary study demonstrated improvement of computed tomography lung imaging and recovery of inflammatory biomarkers	[115]
CR3022	mAbs	Binds specifically to SARS-CoV-2 receptor-binding domain	Cross-reactive antibodies, to be given alone or in combination with other neutralizing antibodies	Not yet, clinically tested in COVID-19 patients	[42,79]
Anti-ACE2 antibody	mAbs	ACE2	Inhibits the interaction between ACE2 and viral S protein, prevent viral invasion into the host	Under development	[116]
Clone 7-508-201	Antibody	Spike protein	Viral entry inhibitor	*In vitro* viral neutralizing activity was completed	[117]
Peptides (P2, P6, P8, and P10)	Peptides	Spike peplomers	Inhibiting oligomerization of S protein and hence preventing viral entry	–	[118]
Adenovirus-vectored vaccine	Whole virus vaccines	Spike protein	Stimulate the immune system to produce antibodies against SARS-Cov-2	Phase III clinical trial demonstrated that a single shot of Janssen COVID-19 vaccine met primary end points with 85% effective in overall protection against COVID-19	[119]
Live-attenuated vaccine	Whole virus vaccines	Multiple viral antigens	Prompts an immune response against COVID-19.	Codagenix and Serum Institute of India developed a nasal covid-19 vaccine (COVI-VAC), Phase I clinical trial is ongoing and initial testing data expected by mid-2021	[120]
Subunit vaccines	Vaccine	Spike protein	Antibodies generated neutralize the viral component, design is based on molecular clamp strategy	It was developed by the University of Queensland and completed Phase I trial. Due to disappointing results vaccine testing was not promoted in Phase II/III clinical trials	[121]
Recombinant subunit-trimer vaccine	S-Trimer vaccine	Trimeric Spike protein	Inhibition of viral entry and specifically neutralize the evaded novel coronavirus virus	Phase I trial depicted promising safety and tolerability profiles and strong neutralizing immune response. Global Phase II/III trial assumed to be initiated in the first half of 2021	[122]
Oral COVID-19 vaccine	Vaccine	Spike protein	Prompts an immune response	Its Phase I trial demonstrated that Vaxart vaccine candidate (VXA-CoV2-1) has triggered various immune responses against COVID-19	[123]
INO-4800	DNA vaccine	Spike protein	Induced host immune response to produce antibodies that block SARS-CoV-2 Spike protein and prevent viral invasion	It has been developed by Inovio in collaboration with Beijing Advaccine Biotechnology. Phase I clinical trial completed and now Phase II clinical trial is ongoing	[124]
CureVac COVID-19 vaccine	mRNA vaccine	–	Provoke immune response against novel coronavirus	Phase III clinical trial is ongoing in Germany	[125]
Non-replicating viral vector	Vaccine	–	Activate the immune system to produce antibodies against SARS-CoV-2	Developed by the University of Oxford in collaboration with AstraZeneca with the efficacy of 82.4%, approved for emergency use (also known as Covishield in India)	[94,126]
Non-replicating viral vector	Vaccine	–	Adenovirus Type 5 Vector provoke a specific immune response	It was developed by CanSino Biological Inc. in collaboration with the Academy of Military Medical Sciences' Institute of Biotechnology, Beijing. Phase III clinical trials are ongoing and approved for limited use and have 65.7% efficacy	[127]
Messenger RNA-based vaccine	mRNA vaccine	–	Produce antiviral proteins in the body	It was developed by a USA-based company Moderna in collaboration with the National Institutes of Health. Currently, it has been approved and has 94.5% efficacy	[128]
Messenger RNA-based vaccine	mRNA vaccine	–	Produce antibodies against SARS-CoV-2	German-based biotech company ‘BioNTech’ in collaboration with Pfizer has developed an mRNA vaccine. It has been approved in several countries and has 95% efficacy	[129,130]
Inactivated virus vaccine	Vaccine	–	Produce strong neutralizing antibody response	It was developed by the Wuhan Institute of Biological Products, its Phased III clinical trial is ongoing, approved for limited use in China and United Arab Emirates	[131,132]
Inactivated virus vaccine	Vaccine	–	Produce neutralizing antibodies against SARS-CoV-2	It was developed by China-based Sinopharm. It has an efficacy of 79.34% approved in China and Bahrain	[133]
Inactivated virus vaccine	Vaccine	–	Produce antibody against SARS-Cov-2	It was developed by a Chinese private company named Sinovac. The developed vaccine named as CoronaVac has been approved in China	[134,135]

### Formulation components of the approved COVID-19 vaccine

The development of commercially effective COVID-19 vaccines is not only dependent on different immunological factors like the nature of the antigen but also primarily impacted by formulation strategy and vaccine delivery aspects. With the advancement in protein- and peptide-based therapeutics, the development of stable and effective vaccines that can withstand the harsh environmental conditions during processing and storage presents a major challenge for formulation scientists. Most of the biological products and vaccines are unstable and liable to degradation during processing and storage. Therefore, several approved formulation ingredients are essential for developing a stable vaccine. Selection of the non-immunogenic excipients and formulation strategy that can protect the critical vaccine components etc., have a huge bearing on the development of novel biological therapeutics [136]. The Pfizer vaccine and Moderna vaccine for COVID-19 are identical in their excipients composition. Both vaccines are developed based on the nanotechnological approach and composed of liposomes that encapsulate mRNA. Liposomes are spherical vesicles composed of bilayer phospholipids. mRNA encapsulated liposomes can be easily taken up by the cells due to their similar composition as the cell membrane. Pfizer and Moderna liposomal vaccines are nearly 100 nm, the same size as that of SARS-COV-2 [137,138]. The detailed formulation aspects of the Pfizer, Moderna liposomal vaccines and other approved vaccines are presented in Table 2.

**Table 2.** Formulation aspects of some of the approved vaccine for COVID-19.

Vaccines	Active ingredients	Excipients	Efficacy	Dose and route of administration	Storage	Ref.
Pfizer (BNT162b2)	Modified mRNA	• (4-hydroxybutyl)azanediyl)bis(hexane-6,1-diyl)bis (ALC-3015) • (2-hexyldecanoate),2-[(polyethylene glycol)-2000]-N,N-ditetradecylacetamide (ALC-0159) • 1,2-distearoyl-snglycero-3-phosphocholine (DPSC) Cholesterol • KCl • KH_2_PO_4_ • NaCl • NaH_2_PO_4_ · 2H_2_O • Sucrose	95%	2 doses, 3 weeks apart, muscle injection	Freezer storage only at -70°C	[138,139]
Moderna (mRNA-1273)	Modified mRNA	• SM-102 • 1,2-dimyristoyl-rac-glycero3-methoxypolyethylene glycol-2000 (PEG2000-DMG) • DSPC Cholesterol • Tris buffer • TRIS hydrochloride • CH_3_COOH • CH_3_COONa • Sucrose	94.5 %	2 doses, 4 weeks apart, muscle injection	30 days with refrigeration, 6 months at -20°C	[137,140]
Bharat Biotech (Covaxin)	Whole-virion inactivated SARS-CoV-2 antigen	• Aluminum hydroxide gel • Imidazoquinolinone • 2-phenoxyethanol • Phosphate buffer saline	78%	2 doses given 4 weeks apart, muscle injection	At least a week at room temperature	[141]
Covishield (Serum Institute of India Ltd)	ChAdOx1 nCoV-19 corona virus vaccine (Recombinant)	• L-Histidine • L-Histidine hydrochloride monohydrate • Magnesium chloride hexahydrate • Polysorbate 80 • Ethanol • Sucrose • Sodium chloride • Disodium edetate dihydrate (EDTA) • Water for injection	76%	2 doses given 4 weeks apart, muscle injection	Stable in the refrigerator for at least 6 months	[142]
Sputnik V	Adenovirus viral vector vaccine	• Tris (hydroxymethyl) aminomethane • NaCl • Sucrose • Magnesium chloride hexahydrate • EDTA sodium salt dihydrate • Polysorbate 80 • Ethanol 95% • Water for injection	91.6%	2 doses given 3 weeks apart, muscle injection	Freezer storage	[143,144]
Janssen Ad26.COV2.S vaccine	Viral vector Ad26.COV2	• Citric acid monohydrate • Trisodium citrate dihydrate • Ethanol • 2-hydroxypropyl-beta-cyclodextrin (HBCD) • Polysorbate 80 • NaCl • NaOH • HCl	72%	Single dose, muscle injection	Up to two years frozen at -4°F (-20°C), and up to 4.5 months refrigerated at 36–46°F (2–8°C).	[145]

In the Pfizer and Moderna vaccine, lipids used for the development of liposomal vaccine carrier that can encapsulate and protect mRNA from degradation. Salts act as a buffer that maintain the pH level close to the pH of the physiological system. Sucrose acts as a cryoprotectant for safeguarding liposomes during freezing.

In Covaxin, imidazoquinolinone has been used as Toll-like receptor-7/8 agonist.

## Conclusion

Innate immunity plays a crucial role in viral clearance and in developing long-term protective immunity for fighting against infectious pathogens in case of a future attack. Thus, harnessing innate immune responses during viral clearance has demonstrated promising outcomes in ongoing clinical trials against COVID-19. Innate immune cells are in constant motion to scan molecular alteration to cells caused by microbial infections throughout the body and present them through antigen-presenting cells to further develop antibodies and long-lasting protective immunity. By targeting these alterations, T-cell responses will be mainly focused on developing long-lasting protective immune cells such as memory T cells and cytotoxic T cells to clear the infected cells. Clinically, the biphasic immune responses initiated during COVID-19 infection may include nonsevere and severe stages. During the primary nonsevere stage, a precise adaptive immune response is a prerequisite for clearing viral infection and stopping its progression to the critical stage. Therefore, strategies for boosting innate immunity at early stages are highly desirable for preventing viral growth and further spread of viral infection. For the development of specific antiviral immunity at both stages of the disease, host genetic background and health status have been demonstrated to be vital in producing long-lasting protective immunity. Whenever there is impairment in protective immune response, innate immunity mediated severe destruction of infected tissue has been noticed. Inflammatory destruction to lung tissues is preferentially mediated by pro-inflammatory macrophages and granulocytes, triggering the development of a life-threatening severe disease stage. Therefore, boosting the innate immune response at the initial stages is needed while suppressing it in the severe stage is life saving. Currently, monoclonal antibody- and convalescent plasma-based immunotherapeutic approaches have conferred potential improvement of clinical symptoms in COVID-19 patients. Hence, harnessing innate immune responses during COVID-19 treatment can be a beneficial strategy to effectively fight against coronavirus.

## Future perspective

For a better understanding of innate immune response during SARS-CoV-2 infection, we need a series of studies that explore the role of innate immunity during the initial and later phase of infection. Additionally, the role of innate immune cells for clearing viral infection and inhibiting the progression of viral infection to severe stages need to be established in COVID-19. Since innate immune cells act as the primary defense system during viral entry and their replication into the host cells, factors or immunogens that can boost the activity of innate immune cells need to be explored. Further, as part of innate immunity, stem cells are found to release antimicrobial peptides that possess antiviral properties. Stem cell activation due to viral infection stimulates the release of antimicrobial peptides, which kills the virus and protect the host from viral infection. Thus, in the future, stem cell therapy may evolve as a viable and highly-effective treatment strategy for fighting against COVID-19 infection.

**Executive summary**

- COVID-19 is an infectious and highly-contagious disease.

**General information about coronaviruses**

- Coronaviruses are the viruses that belong to the Coronaviridae family and *Betacoronavirus* genus, which mainly infect mammals and some birds.

**Role of innate immunity in COVID-19**

- Innate immunity portrays a key role in clearing invading viruses and developing long-term adaptive immunity for future events.
- The immunotherapy strategy has a great potential to develop a vaccine against SARS-CoV-2 in the coming future.
- The role of harnessing innate immunity in clearing viral infection, generally based on the principle of enhancing the T-cell responses by targeting activating or inhibitory immune checkpoints pathways.

**Mechanism of innate immunity during viral infection**

- The activity of innate immunity largely depends on the involvement of a wide variety of cells that include antigen-presenting cells such as dendritic cells, white blood cells, mast cells, lymphoid cells and natural killer cells.
- Innate immunity is in constant motion to scan molecular alteration to cells caused by microbial infections throughout the body.

**Future perspective**

- Hence, harnessing innate immune responses during COVID-19 treatment can be a beneficial strategy to fight against coronavirus.
